# Astragaloside IV prevents calpain-1-mediated cardiac hypertrophy and fibrosis induced by diabetes

**DOI:** 10.3389/fcvm.2026.1670472

**Published:** 2026-02-05

**Authors:** Qinglong Zhang, Ning Zhao, Silin Wei, Meili Lu, Kangyin Chen

**Affiliations:** 1 Department of Cardiology, Tianjin Key Laboratory of Ionic-Molecular Function of Cardiovascular Disease, Second Hospital of Tianjin Medical University, Tianjin Institute of Cardiology, Tianjin, China; 2 Internal Medicine-Cardiovascular Department, The First Affliated Hospital of Jinzhou Medical University, Jinzhou, China; 3 Department of Biochemistry and Molecular Biology, Basic Medical College, Jinzhou Medical University, Jinzhou, Liaoning, China; 4 Key Laboratory of Cardiovascular and Cerebrovascular Drug Research of Liaoning Province, Jinzhou Medical University, Jinzhou, China

**Keywords:** apoptosis, astragaloside IV, Ca^2+^ overload, calpain-1, oxidative stress

## Abstract

**Objective:**

Astragaloside IV (AsIV) has been reported to alleviate diabetes-induced endothelial dysfunction by inhibiting calpain-1. This study aimed to determine whether the same mechanism underlies its protective effect against diabetic cardiomyopathy (DCM).

**Methods:**

At the *in vivo* level, calpain-1 knockout mice with the genotype Capn1 EK684−/− (Capn1 EK684 knockout mice) were used to establish a type 2 diabetic cardiomyopathy model. At the *in vitro* level, H9c2 cells and cardiac fibroblasts were stimulated with high glucose to construct corresponding models. Meanwhile, a calpain-1 overexpression lentivirus was constructed to assess the effect of calpain-1 on myocardial cell injury. Different doses of AsIV were then used to intervene in diabetic mice and H9c2 cells. Body weight, blood glucose, myocardial hypertrophy, myocardial fibrosis, cardiac function, Ca^2+^ overload and its regulation, myocardial cell apoptosis and oxidative stress were evaluated in the current study.

**Results:**

AsIV could not completely normalize blood glucose in mice, but could significantly improve cardiac systolic and diastolic function, myocardial hypertrophy and fibrosis. The beneficial effect of calpain-1 gene knockout on diabetic cardiomyopathy was similar to that of AsIV, and calpain-1 knockout did not further enhance the beneficial effect of AsIV. Calpain-1 overexpression abolished the beneficial effect of AsIV on high glucose induced H9c2 cell injury and fibroblast proliferation. In addition, the intracellular Ca^2+^ overload, abnormal levels of sarco/endoplasmic reticulum Ca^2+^-ATPase 2a (SERCA2a), phosphorylation of phospholamban (p-PLN) and ryanodine receptor 2 (p-RyR2), apoptosis and oxidative stress associated with DCM were also improved by AsIV or calpain-1 knockout, and AsIV has the capacity to suppress the overactivation of calpain-1 and calcium/calmodulin-dependent protein kinase Ⅱ (CaMKII).

**Conclusions:**

AsIV could ameliorate intracellular Ca^2+^ overload, apoptosis, and oxidative stress by regulating the calpain-1/CaMKII pathway, thereby improving myocardial hypertrophy and fibrosis caused by diabetes mellitus.

## 1 Introduction

Hyperglycemia is a hallmark of diabetes, a metabolic disorder that has emerged as a major public health issue in the 21st century. Estimates from the International Diabetes Federation suggest that by 2040, the global prevalence of diabetes will rise to 642 million adults (1). Chronic elevated blood glucose levels can cause extensive organ damage, with cardiovascular disease being a prominent consequence. According to the expert consensus statement of the Heart Failure Association (HFA) of the European Society of Cardiology (ESC) and the ESC Working Group on Myocardial & Pericardial Diseases, diabetic myocardial disorder-previously termed diabetic cardiomyopathy (DCM)-is defined as systolic and/or diastolic myocardial dysfunction in patients with diabetes (2). In this disorder, systemic hyperglycemia, insulin resistance, and renin-angiotensin-aldosterone system (RAAS) activation converge in the heart, inducing oxidative stress, advanced glycation end products (AGEs) accumulation, and lipotoxicity. These pathological processes, together with Ca^2+^ dysregulation, myocardial hypertrophy, and stiffening of titin isoforms, precipitate progressive diastolic dysfunction and eventual systolic dysfunction (3, 4). Thus, developing effective therapeutic strategies—particularly pharmacological interventions—to prevent and manage diabetic cardiomyopathy is of paramount importance for individuals with diabetes.

Calpains constitute a family of calcium-dependent intracellular cysteine proteases that play crucial roles in diverse cellular functions. These proteases mediate the cleavage of a wide range of protein substrates, thereby influencing processes such as cell proliferation, division, oxidative stress response, and apoptosis (5, 6). The two primary isoforms, calpain-1 and calpain-2, exhibit Ca^2+^-dependent partial proteolysis activity. Both calpain-1 and calpain-2 consist of distinct large 80-kDa catalytic subunits. The capn1 gene encodes the catalytic subunit of calpain-1, whereas the catalytic subunit of calpain-2 is encoded by the capn2 gene. Additionally, they share a common small 28-kDa regulatory subunit encoded by the capn4 gene. Calpain-1 is ubiquitously expressed and has been implicated in cardiopulmonary vascular disease injury due to a variety of stressors and the progression of heart failure (7–9). Viral myocarditis caused by Coxsackievirus B3 (CVB3), calpain-1 accumulates in mitochondria, which can hydrolyze ATP5A to produce ROS, and then lead to myocardial pyroptosis and necrosis (10). As a second messenger, intracellular Ca^2+^ is closely related to the activation of a variety of signaling pathways related to myocardial hypertrophy and fibrosis. Calpain-1 inhibition with small interfering RNA transfection inhibited Ca^2+^ overload and apoptosis, and attenuated isoproterenol-induced H9c2 cardiomyocytes hypertrophy (11). Previous studies have demonstrated that calpain-1 is involved in the development and progression of diabetic cardiomyopathy. These findings suggest that calpain-1 could be a promising therapeutic target for mitigating cardiac complications in diabetes. Selective deletion of calpain with endothelial cell in mice increased the protein levels of β-catenin, inhibited apoptosis, improved angiogenesis, and subsequently alleviated diabetic cardiomyopathy (12). Our team has constructed calpain-1 knockout mice in previous studies, and confirmed that calpain-1 inhibition can improve endothelial dysfunction induced by high glucose and hypoxia, but there is no clear evidence whether it can alleviate diabetic cardiomyopathy.

Astragaloside Ⅳ (AsIV) is one of the main active ingredients of Astragalus membranaceus, which has anti-inflammatory, anti apoptotic, antioxidant and antiviral pharmacological effects (13–16). AsⅣ and its derivative HHQ16 improved infarction-induced myocardial injury and cardiac dysfunction, which effect was related with improving mitochondrial morphology and function, regulating energy metabolism, reducing inflammation of infarcted heart tissue and denigrating Egr2-affiliated transcript lnc9456 in the heart (17–19). In addition, AsIV can improve a variety of complications caused by diabetes, including diabetic nephropathy, diabetic encephalopathy, and vascular disease, etc. (20–22). In the rat model of diabetes, AsⅣ can improve diabetic cardiomyopathy by regulating lipid metabolism and energy metabolism (23). We have previously discussed the effects of AsIV on myocardial hypertrophy, pulmonary hypertension and endothelial injury (24–26), and these effects are related to the regulation of calpain-1. However, whether AsⅣ improves diabetic cardiomyopathy by regulating Ca^2+^ and calpain-1 remains unclear.

## 2 Materials and methods

### 2.1 Experimental animal

**Figure 1:**
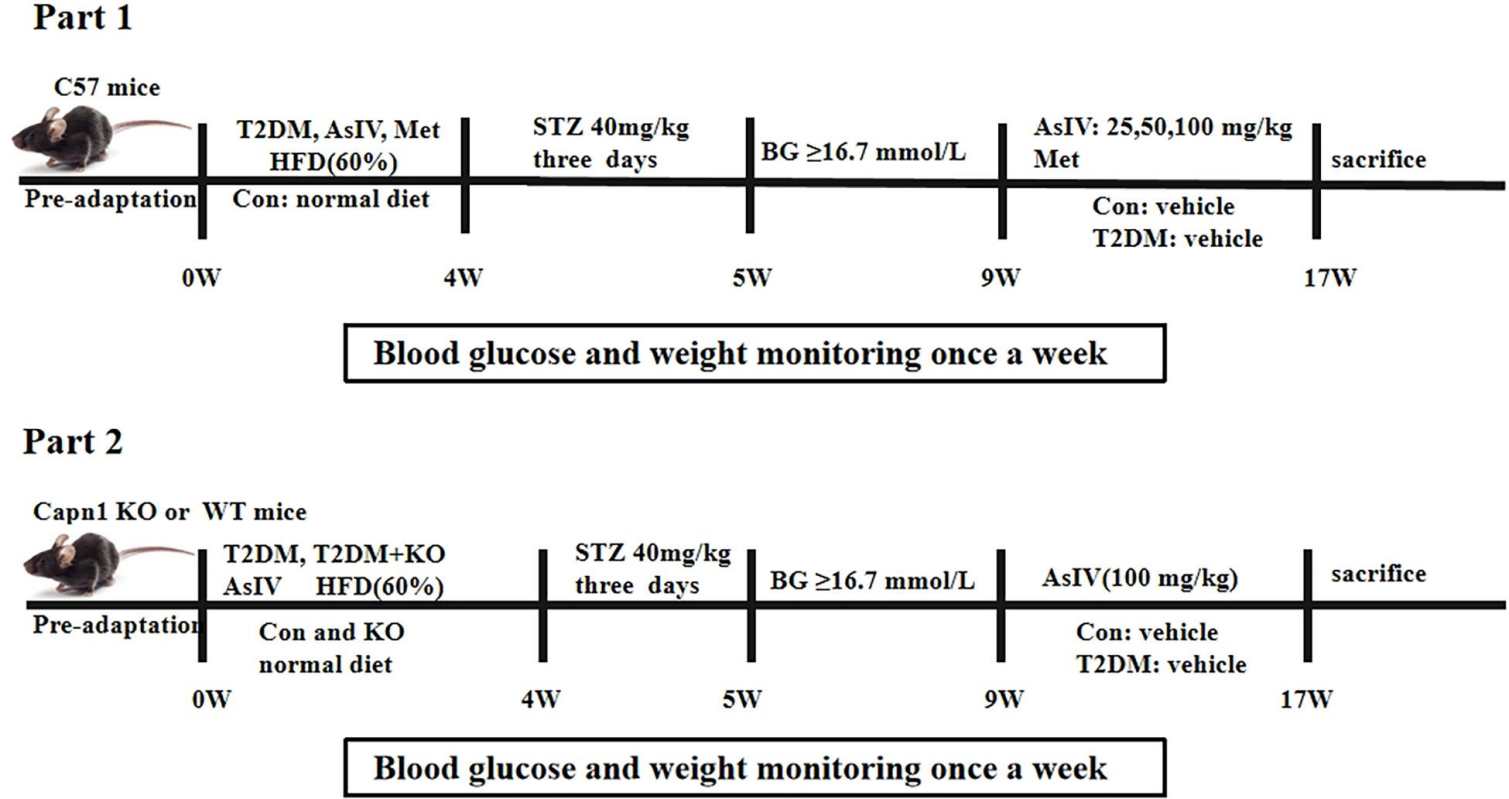
Animal treatment schedules: part 1 and part 2.

All animal experiments were conducted with the approval of the Animal Care and Use Committee at Jinzhou Medical University (20250018). Capn1 EK684−/− mice and the same background C57BL/6N mice were purchased from Cyagen Biosciences co., Ltd. After one week of adaptive feeding, 7–8 week male C57BL/6N mice (20–25 g) were stratified by body weight and then randomly allocated to control or high-fat diet group using a computerized random-number generator; assignments were concealed in sealed envelopes and revealed by an investigator not involved in outcome assessment. All subsequent experiments and analyses were performed blinded to group allocation. After a high-fat diet for 4 weeks, mice were injected intraperitoneally with streptozotocin (STZ) at a dose of 40 mg/kg for three consecutive days, and the STZ was dissolved in 0.1% citric acid solution. The blood sugar levels of the mice were measured one week later. Mice with blood sugar levels higher than 16.7 mmol/L were identified as type 2 diabetic mice (27). According to the requirements of the experimental purpose, this animal experiment was divided into two parts. The first part was divided into six groups (*n* = 10): normal group (Con), type II diabetes mellitus model group (T2DM), T2DM + low-dose AsIV group (25 mg/kg), T2DM + medium-dose AsIV group (50 mg/kg), T2DM + high-dose AsIV group (100 mg/kg), and T2DM + metformin group. The second part was divided into six groups (*n* = 10): normal group (Con), calpain-1 knockout group (KO), model group (T2DM), T2DM + KO group, T2DM + AsIV group, and T2DM + AsIV + KO group. AsIV was dissolved in a 0.5% sodium carboxymethyl cellulose solution and administered orally to the mice for 8 consecutive weeks. During the entire experimental duration, the tail vein blood glucose levels and body weight of all mice were assessed weekly until the study was completed (Figure 1).

### 2.2 Echocardiographic examination

At the end of the experiment, mice (*n* = 4 per group, predetermined by an a-priori power analysis; G*Power 3.1.9.7, Cohen's d = 1.6, power = 0.80, α = 0.05) were anesthetized with 2% isoflurane and examined with the Sigma VET system (Esaote, Italy; SL3116 matrix probe). Investigators performing the scans and subsequent analyses were blinded to treatment allocation; animal order was randomized (stratified by body weight, sealed-envelope method) and cage labels concealed during imaging. Long-axis M-mode recordings were used to measure left ventricular internal dimension in diastole(LVIDd), left ventricular internal dimension in systole (LVIDs), left ventricular ejection fraction(LVEF), left ventricular fractional shortening(LVFS) and heart rate(HR). Pulsed-wave Doppler in the apical four-chamber view provided peak early (E) and late (A) mitral inflow velocities for E/A ratio calculation.

### 2.3 H&E and masson staining

Fresh cardiac tissues were first fixed in 4% paraformaldehyde for 24 h, followed by paraffin embedding and sectioning. The sections were subsequently dewaxed, rehydrated, and stained with hematoxylin and eosin. Finally, images of the stained sections were obtained using a light microscope.

Heart tissue sections were first deparaffinized and then dehydrated using a series of graded alcohols. Subsequently, Masson staining was applied to evaluate collagen accumulation in these sections. The sections stained with Masson were examined under a Leica DMI 3000B light microscope to assess structural alterations and the extent of fibrosis in the cardiac tissue.

### 2.4 H9c2 cells culture and intervention

The culture medium was changed every two days and passed after the H9c2 cells reached a confluence state at the bottom of the culture vessel. After another 24 h of incubation, relevant treatment factors were added. H9c2 cells were cultured in Dulbecco's Modified Eagle Medium (DMEM) with normal glucose (5.5 mmol/L) or high glucose (50 mmol/L). Different doses of Astragaloside IV (20, 40, and 80 μmol/L) and MDL-28170 (20 μmol/L) were added to the treatment groups, and the cells were collected for subsequent experimental treatment after continued cultivation for 48 h. H9c2 cells were transduced using lentivirus, and the transfection efficiency was observed under fluorescence microscope. Cells that successfully overexpressed calpain-1 were treated with Astragaloside IV (80 μmol/L), KN93 (10 μmol/L) or KN92 (10 μmol/L). Cells transfected with lentivirus without calpain-1 gene served as the control group (Con group).

### 2.5 DHE for ROS analysis and enzyme-linked immunosorbent assay

Dihydroethidium (DHE) staining was applied to heart tissues and cells for reactive oxygen species (ROS) detection. After staining, the samples were incubated in the dark at 37 degrees Celsius for 30 min, then washed with phosphate buffered saline (PBS) for 5 min, and then images of the stained samples were taken using a fluorescent microscope.

The levels of malondialdehyde(MDA), superoxide dismutase(SOD) and glutathione(GSH) in blood and cell supernatant of cells were measured using enzyme-linked immunosorbent assay (ELISA). The enzyme-linked immunosorbent assay was performed in accordance with the instructions provided by the manufacturer.

### 2.6 Immunohistochemistry and immunofuorescence staining

Paraffin-embedded heart tissue was cut into sections with a thickness of 5 μm. These sections were then subjected to deparaffinization and rehydration through a series of graded ethanol and xylene treatments. Finally, the sections were mounted using neutral balsam and covered with coverslips. For antigen retrieval, sections were sliced and blocked with goat serum for 1 h at room temperature. The phospho-ryanodine receptor 2(p-RyR2) antibody (1:100) was applied and incubated overnight at 4°C. Following washing, the sections were incubated at room temperature for 1.5 h with horseradish peroxidase-conjugated goat anti-mouse/rabbit IgG. The chromogenic reaction was carried out using a diaminobenzidine (DAB) kit. Finally, tissue morphology was observed under a microscope, and images were captured.

In preparation for immunofluorescence analysis, H9c2 cells were first fixed with 4% paraformaldehyde and then washed three times with PBS for 5 min each. After this, cells were treated with 0.1% Triton X—100 for 10 min to promote permeabilization, and then incubated with 5% bovine serum albumin (BSA) for 1 h to block non-specific binding sites. Next, the cells were incubated with the primary anti-calpain- 1 antibody (diluted 1:100) at 4°C overnight. After completing this step, stain the cell nuclei with DAPI for 5 min. The final step is to image the cells using fluorescence microscopy.

### 2.7 Culture and treatment of myocardial fibroblasts

Cardiac fibroblasts were obtained from neonatal rats. After disinfection, the hearts of 1-to 3-day-old rats were excised and cut into small pieces. The minced tissue is subjected to trypsin digestion. The resulting cell suspension was centrifuged and then resuspended in DMEM supplemented with 15% fetal bovine serum. The cells were then seeded into culture flasks and incubated in a cell incubator for 2 h to promote fibroblast attachment. Once the cells reached 80% to 90% confluence, subculture was performed with 0.25% trypsin. For the experimental flow, the third generation fibroblasts were selected and cultured in DMEM containing normal glucose (5.5 mmol/L) or high glucose (50 mmol/L). Treatment groups were exposed to different concentrations of AsIV (20, 40, 80 μmol/L) and MDL-28170 (20 μmol/L). After a 48 h incubation period, cells were collected for subsequent experimental analysis.

### 2.8 CCK-8 proliferation assay

Cell viability under different conditions was evaluated using CCK-8 kits. According to the experimental groups, the corresponding drug concentrations were added to the cells. After a 24 h incubation, the cells were treated with 10 μL of CCK-8 solution for another 2 h. Finally, the absorbance was measured to assess cell viability.

### 2.9 Ca^2+^ staining

Cells are first cultured on 24 well culture dishes and washed with PBS to remove residual medium. A fluorescent Ca^2+^ indicator Fluo-3/AM probe is then prepared according to the kit instructions. For adherent cells, the staining solution is applied to the cells, which are then incubated in the dark for 30 min. Following incubation, the staining solution is discarded and the cells are rinsed with PBS. Following staining, cells are observed under a fluorescence microscope.

### 2.10 Edu staining

To examine cell proliferation, H9c2 cells were harvested and cultured in a well plate. The cells were then treated according to the experimental requirements. Subsequently, EdU kit (Beyotime) was utilized to detect cell proliferation. The experiment was conducted strictly following the guidelines provided by the manufacturer of the EdU kit.

### 2.11 Flow cytometry analysis

Apoptosis was assessed using the Annexin V-FITC Apoptosis Detection Kit, following the manufacturer's protocol. H9c2 cells were seeded in well plates and exposed to the designated treatment for 24 h. Subsequently, the H9c2 were harvested, washed three times with PBS, and stained with Annexin V-FITC solution for 15 min in the dark.

### 2.12 Phalloidin and JC-1 staining

For phalloidin and JC-1 staining, H9c2 cells was divided into different groups and incubated with respective reagents. Subsequently, the cells were processed following the staining protocols for phalloidin (C2207S, Beyotime Biotechnology) and JC-1 (C2006, Beyotime Biotechnology).

### 2.13 Molecular docking

Molecular docking analysis was conducted using AutoDock (version 4.2.6)-Vina to explore the binding interactions between Astragaloside IV and the proteins calpain-1, SERCA2a, and RyR2. The chemical structure of Astragaloside IV was retrieved from the PubChem database (https://pubchem.ncbi.nlm.nih.gov), while the protein structures were obtained from the AlphaFold Protein Structure Database (https://alphafold.com). The docking simulations were performed using AutoDock-Vina, and the interactions between the small molecule and proteins were visualized and analyzed using PyMOL (version 2.6).

### 2.14 Western blot

Protein extraction from heart tissues and H9c2 cells was conducted using RIPA buffer. The concentration of the extracted proteins was determined using the BCA Protein Assay Kit. Following this, equal quantities of protein were subjected to SDS-PAGE and subsequently transferred to nitrocellulose membranes. The membranes were blocked at room temperature for 1.5 h using a 5% milk solution in TBST, followed by incubation with the corresponding primary antibodies at 4℃ overnight. B-cell lymphoma 2 (Bcl-2, Cat. No. 26593-1-AP, 1:3 000, Proteintech), Bcl-2-associated X protein (Bax, Cat. No. 50599-2-Ig, 1:4 000, Proteintech), calpain-1 (Cat. No. 10538-1-AP, 1:10 000, Proteintech), sarco/endoplasmic reticulum Ca^2+^-ATPase 2a (SERCA2a, Cat. No. 27311-1-AP, 1:5 000, Proteintech), phosphorylated phospholamban at threonine 17 (p-PLN-T17, Cat. No. AP0910, 1:5 000, ABclonal), phospholamban (PLN, Cat. No. A17964, 1:5 000, ABclonal), Ca^2+^/calmodulin-dependent protein kinase II (CaMKII, Cat. No. 13730-1-AP, rabbit polyclonal, 1:5 000, Proteintech), phosphorylated CaMKII at threonine 286 (p-CaMKII-T286, Cat. No. AP0255, 1:5 000, ABclonal), transforming growth factor beta-1 (TGF-β1, Cat. No. A2124, 1:5 000, ABclonal), Smad2 (Cat. No. A11498, 1:1 000, ABclonal), cleaved caspase-3 (Cat. No. A28349, 1:500, ABclonal), caspase-3 (Cat. No. A11319, 1:500, ABclonal), atrial natriuretic peptide (ANP, Cat. No. A14755,1:500, ABclonal) and B-type natriuretic peptide (BNP, Cat. No. A2179, 1:1 000, ABclonal) were detected by western blot. On the following day, the membrane was incubated with the corresponding HRP-conjugated secondary antibody for 1.5 h. Subsequently, the membrane was washed three times with TBST, and the immune complexes were visualized using the ECL chemiluminescent substrate.

### 2.15 Statistics

SPSS 23.0 statistical software was used for data management, and all data were expressed as mean ± standard deviation (SD). GraphPad Prism (version 8.0.2) was applied for statistical analysis and graph plotting. For group comparisons, one-way analysis of variance (one-way ANOVA) was used to test the overall differences among groups first. After confirming the overall significance, Tukey's *post hoc* test was further performed for pairwise comparisons. To control potential type I errors caused by multiple comparisons, the Bonferroni correction was adopted, with the overall alpha level set at 0.05. *p* < 0.05 was considered statistically significant.

## 3 Results

### 3.1 Blood glucose and weight of diabetes mice

To induce a type 2 diabetes model, mice were fed a high-fat diet for 4 weeks, followed by intraperitoneal injection of STZ at a dose of 40 mg/kg for three consecutive days. At the 9th week of modeling, mice were treated with AsIV and metformin, and relevant indicators were measured after continuous administration for 8 weeks. After the experiment, compared with the normal group, the mice in the T2DM group showed a significant decrease in body weight, an increase in water intake, less movement, curly hair, and no luster (Supplementary Figure S1). Compared with the model group, the mice in the middle and high doses of AsIV and metformin groups showed increase in body weight, decrease in water intake, shiny hair, and tendency to move more. Although AsIV reduced the blood glucose level in the model group, it did not completely normalized the blood glucose level of mice, and metformin could significantly reduce the blood glucose level of diabetes mice, suggesting that the improvement of diabetes symptoms by AsIV was independent of the regulation of blood glucose (Table 1).

**Table 1:** Body weight and blood glucose of C57BL/6 mice.

Groups	Body weight (g)				Blood glucose level (mmol/L)			
	0 W	4 W	9 W	17 W	0 W	4 W	9 W	17 W
Con	20.88 ± 0.99	24.00 ± 0.93	28.13 ± 2.42	32.25 ± 2.12	5.08 ± 0.54	5.33 ± 0.57	5.24 ± 0.34	5.49 ± 0.43
T2DM	21.38 ± 1.41	28.00 ± 2.83*	29.50 ± 2.88	25.88 ± 2.10**	5.35 ± 0.37	5.15 ± 0.16	22.48 ± 3.46**	22.56 ± 2.91**
T2DM + AsIV (L)	20.75 ± 1.75	27.25 ± 2.25	30.50 ± 3.07	26.75 ± 2.55	5.33 ± 0.41	5.00 ± 0.41	23.56 ± 4.78	20.78 ± 3.62
T2DM + AsIV (M)	21.00 ± 1.60	28.13 ± 2.10	30.00 ± 1.85	28.38 ± 2.62^#^	5.39 ± 0.56	5.36 ± 0.40	22.06 ± 1.82	18.06 ± 4.12^#^
T2DM + AsIV (H)	21.88 ± 1.55	28.38 ± 1.60	30.88 ± 2.80	29.25 ± 1.28^#^	5.39 ± 0.32	5.16 ± 0.33	23.49 ± 4.98	16.56 ± 3.89^#^
T2DM + Met	21.25 ± 1.83	28.36 ± 2.62	29.75 ± 2.19	28.63 ± 1.77^#^	5.46 ± 0.59	5.24 ± 0.68	22.91 ± 3.07	9.36 ± 1.84^#^

The data were presented as the mean ± SD.

* *p* < 0.05.

** *p* < 0.01 vs. Con group.

# *p* < 0.05 vs. T2DM group.

### 3.2 Astragaloside IV improves cardiac injury induced by diabetes

**Figure 2:**
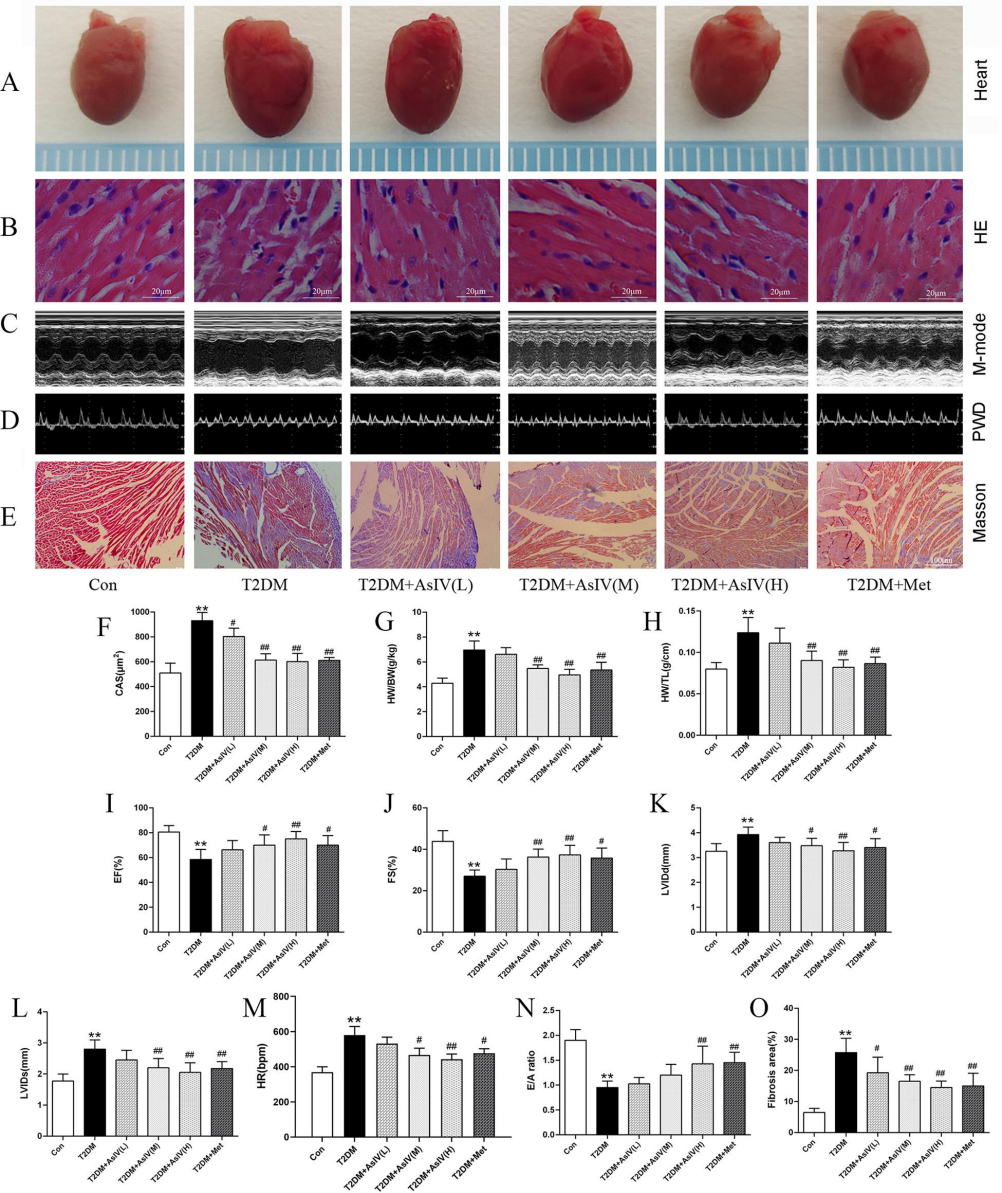
Astragaloside IV mitigates cardiac injury induced by diabetes. **(A)** The hearts of different groups. **(B)** HE staining of heart tissue. **(C)** M-model of different hearts. **(D)** PWD of different hearts. **(E,O)** Masson staining and fibrosis area of heart tissue. **(F)** CAS according to HE staining. **(G,H)** HW/BW and HW/TL of different groups. **(I–M)** Left ventricular EF, FS, LVIDd, LVIDs and HR according to M-mode. **(N)** E/A of diastolic mitral flow velocities. The data were presented as the mean ± SD (*n* = 8 for G and H and *n* = 4 for others). * *p* < 0.05, **p < 0.01 vs. Con group; ^#^*p* < 0.05, ^##^*p* < 0.01 vs. T2DM group.

Diabetic cardiomyopathy was successfully established, with increased HW/BW and HW/TL ratio, and a 1.83 ± 0.13 fold increase in cross section area. HE stating showed irregular fibre arrangement, broken nuclear membranes and inflammatory cell infiltration in T2DM group. Treatment with AsIV and metformin had no significant effect on heart weight, but prevented cardiac hypertrophy, manifested by decreased cross section area, HW/BW and HW/TL ratio, and improved morphological changes (Figures 2A,B,F–H). In addition, Masson staining results showed significant collagen deposition and 3.96 ± 0.70 fold fibrosis area in the ventricular tissue of the model group mice, which was improved in the AsIV and metformin groups (Figures 2E,Q). In addition, The results of echocardiography showed that the cardiac cavity of diabetic mice was enlarged and the systolic function was impaired, which showed as increased LVIDd and LVIDs, and decreased LVEF and LVFS (Figures 2I–L). AsIV and metformin can partially reverse the impaired systolic function and reduce left ventricular enlargement. Meanwhile, mitral valve E/A was decreased and HR was increased in T2DM group, suggesting impaired diastolic dysfunction. The intervention of AsIV could improve the diastolic function of diabetes mellitus to a certain extent, which is manifested by increased E/A and decreased HR (Figures 2D,M,N). These results suggest that AsIV can mitigate myocardial hypertrophy and fibrosis, and improve cardiac dysfunction in diabetes cardiomyopathy to a certain extent.

### 3.3 Astragaloside IV prevents oxidative stress and apoptosis induced by diabetes

**Figure 3:**
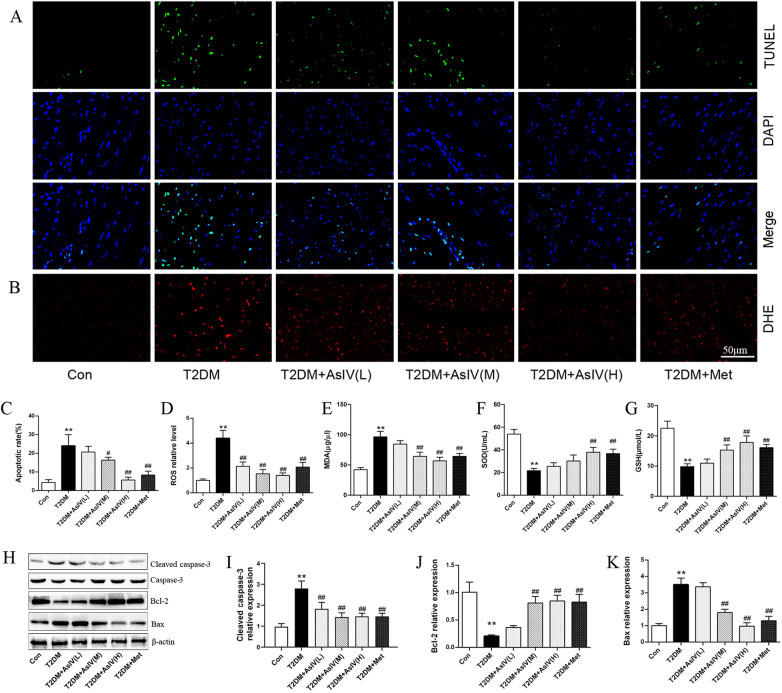
Astragaloside IV prevents oxidative stress and apoptosis induced by diabetes. **(A,C)** TUNEL staining and apoptotic rate. **(B,D)** DHE staining and ROS level. **(E–G)** The content of MDA, SOD and GSH in serum. **(H–K)** The protein expression of Bax, Bcl-2, cleaved caspase-3 and caspase-3. The data were presented as the mean ± SD (*n* = 4 for A-D, *n* = 8 for **E–G**, and *n* = 3 **H–K**). *p < 0.05, **p < 0.01 vs. Con group; ^#^*p* < 0.05, ^##^*p* < 0.01 vs. T2DM group.

Cardiac hypertrophy, apoptosis, and oxidative stress form a vicious cycle that exacerbates myocardial injury and ultimately leads to decompensation of cardiac function. The content of ROS, MDA and SOD were measured as indexes of oxidative stress, and TUNEL staining and expression of Bcl-2, Bax, and cleaved caspase-3 were measured as as indexes of apoptosis. Versus the Con group (4.3% ± 1.5%), the TUNEL positive cells in the myocardial tissue of T2DM group elevated significantly, with an apoptosis rate of 24.0% ± 6.0%, while the TUNLE positive cells in the high dose of AsIV group and metformin group decreased substantially, with apoptosis rates of 5.7% ± 1.5% and 8.3% ± 2.1%, respectively (Figures 3A,C). The WB results showed that compared with the model group, AsIV upregulated the expression of anti apoptotic gene Bcl-2 and decreased the expression of apoptosis inducing gene Bax, and cleaved caspase-3 (Figures 3H–K). The ROS in the myocardial tissue of the T2DM group mice increased significantly, the MDA content increased, and the SOD level decreased. As compared with the T2DM group, the AsIV group showed improvement in oxidative stress indicators (Figures 3D–G).

### 3.4 Astragaloside IV regulates calpain-1 and Ca^2+^ homeostasis

**Figure 4:**
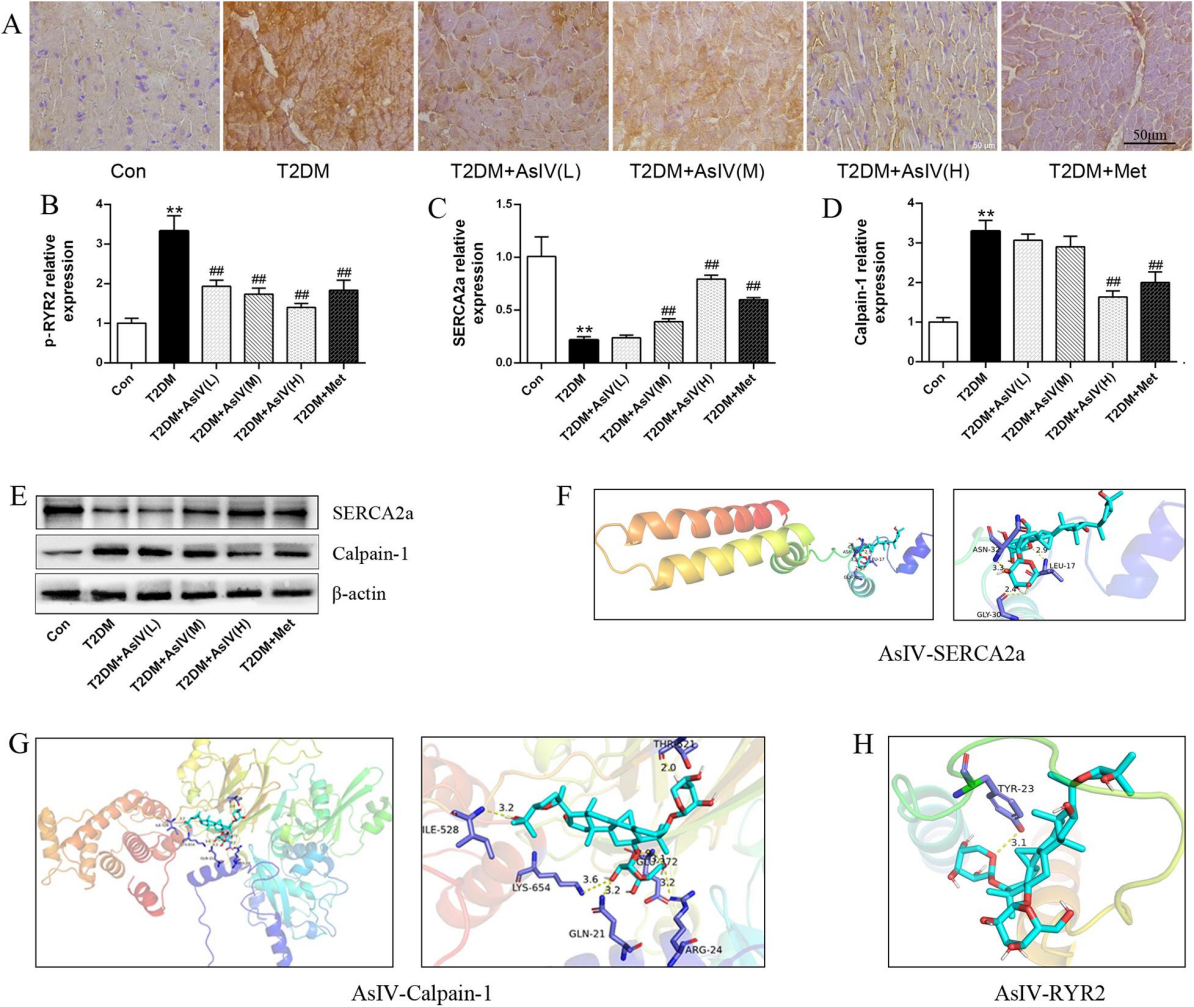
Astragaloside IV regulates calpain-1 and Ca^2+^ homeostasis. **(A,B)** Immunohistochemical staining and relative expression of p-RyR2. **(C–E)** The protein expression of calpain-1 and SERCA2a. **(F–H)** Molecular docking results of AsIV with calpain-1, RyR2 and SERCA2a. The data were presented as the mean ± SD (*n* = 3). *p < 0.05, **p < 0.01 vs. Con group; ^#^*p* < 0.05, ^##^*p* < 0.01 vs. T2DM group.

Abnormal Ca^2+^ homeostasis and activation of calpain-1 contribute to diabetic cardiomyopathy. As shown in Figure 4, the T2DM group displayed boosted expression of p-RyR2 (T2DM group: 3.33 ± 0.38; Con group:1.00 ± 0.13) and lessened expression of SERCA2a (T2DM group: 0.22 ± 0.03; Con group:1.00 ± 0.19) relative to the control group. Various interventions were found to modulate these protein expressions to different extents. Specifically, they could decrease p-RyR2 expression and enhance SERCA2a expression (Figures 4A–C). Notably, the high-dose AsIV group demonstrated a significantly more pronounced effect compared to metformin treatment. Similarly, relative to the model group, high-dose AsIV significantly inhibited the increased expression of calpain-1 and had a stronger effect than metformin (Figures 4D,E). In addition, The molecular docking results showed that AsIV stably interacted with calpain-1, RyR2 and SERCA2a with docking energies lower than −5 kcal/mol (Figures 4F–H). These results suggest that AsIV ameliorates diabetic cardiomyopathy, at least in part, by inhibiting calpain-1 overactivation, thereby restoring intracellular Ca^2+^ homeostasis through normalization of RyR2 and SERCA2a function.

### 3.5 Astragaloside IV attenuates cardiac hypertrophy and fibrosis by inhibiting calpain-1 in diabetic mice

**Figure 5:**
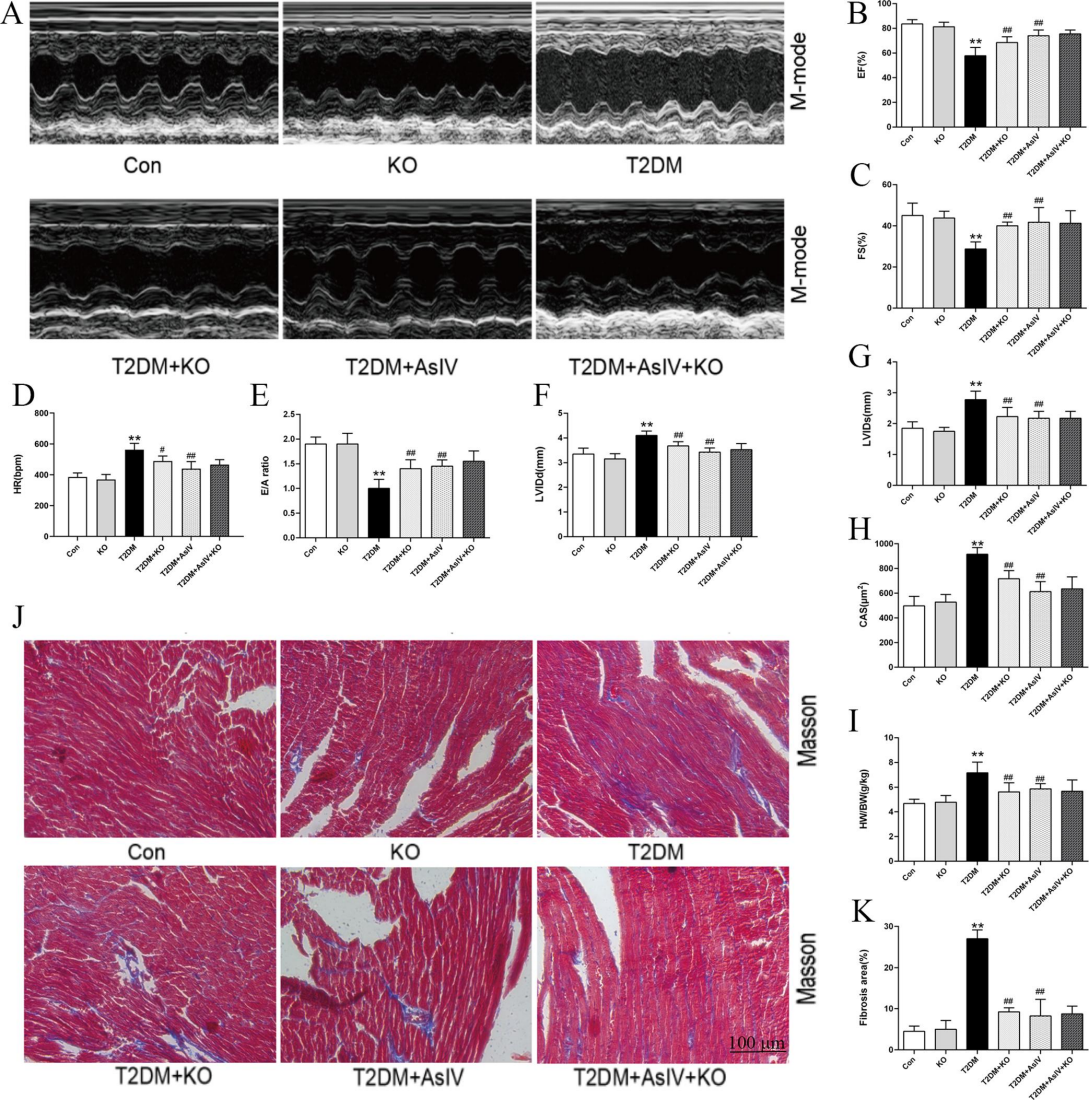
Astragaloside IV attenuates cardiac hypertrophy and fibrosis by inhibiting calpain-1 in diabetic mice. **(A)** M-model of different hearts. **(B,C)** Left ventricular EF and FS. **(D)** HR of different groups. **(E)** E/A of diastolic mitral flow velocities. **(F,G)** Left ventricular LVIDd and LVIDs. **(H)** CAS according to HE staining. **(I)** HW/BW of different groups. **(J,K)** Masson staining and fibrosis area. The data were presented as the mean ± SD (*n* = 8 for I, and *n* = 4 for others). *p < 0.05, **p < 0.01 vs. Con group; ^#^*p* < 0.05, ^##^*p* < 0.01 vs. T2DM group.

In order to further clarify whether AsIV can improve diabetes cardiomyopathy by inhibiting calpain-1, the current study analyzed the effect of AsIV at the level of calpain-1 gene knockout mice. Compared with mice in the T2DM group, calpain-1 knockout mice showed increased body weight and a slight reduction in blood glucose (Supplementary Figure S2). The results of echocardiography showed calpain-1 knockout improved the systolic and diastolic function of the heart, and its effect is similar to that of AsIV (Figures 5A–G). After intervention with AsIV in calpain-1 knockout mice, there was no further increase in the improvement effect of AsIV compared to the AsIV alone group. Similarly, hypertrophy and fibrosis indicators showed that calpain-1 knockout improved myocardial hypertrophy and fibrosis induced by diabetes, but there was no further enhancement effect when combined with AsIV (Figures 5H–J). These results indicated that the improvement effect of AsIV on cardiac hypertrophy and fibrosis is achieved via calpain-1.

### 3.6 Astragaloside IV alleviates oxidative stress, apoptosis and Ca^2+^ metabolism imbalance through inhibiting calpain-1 in diabetes mice

**Figure 6:**
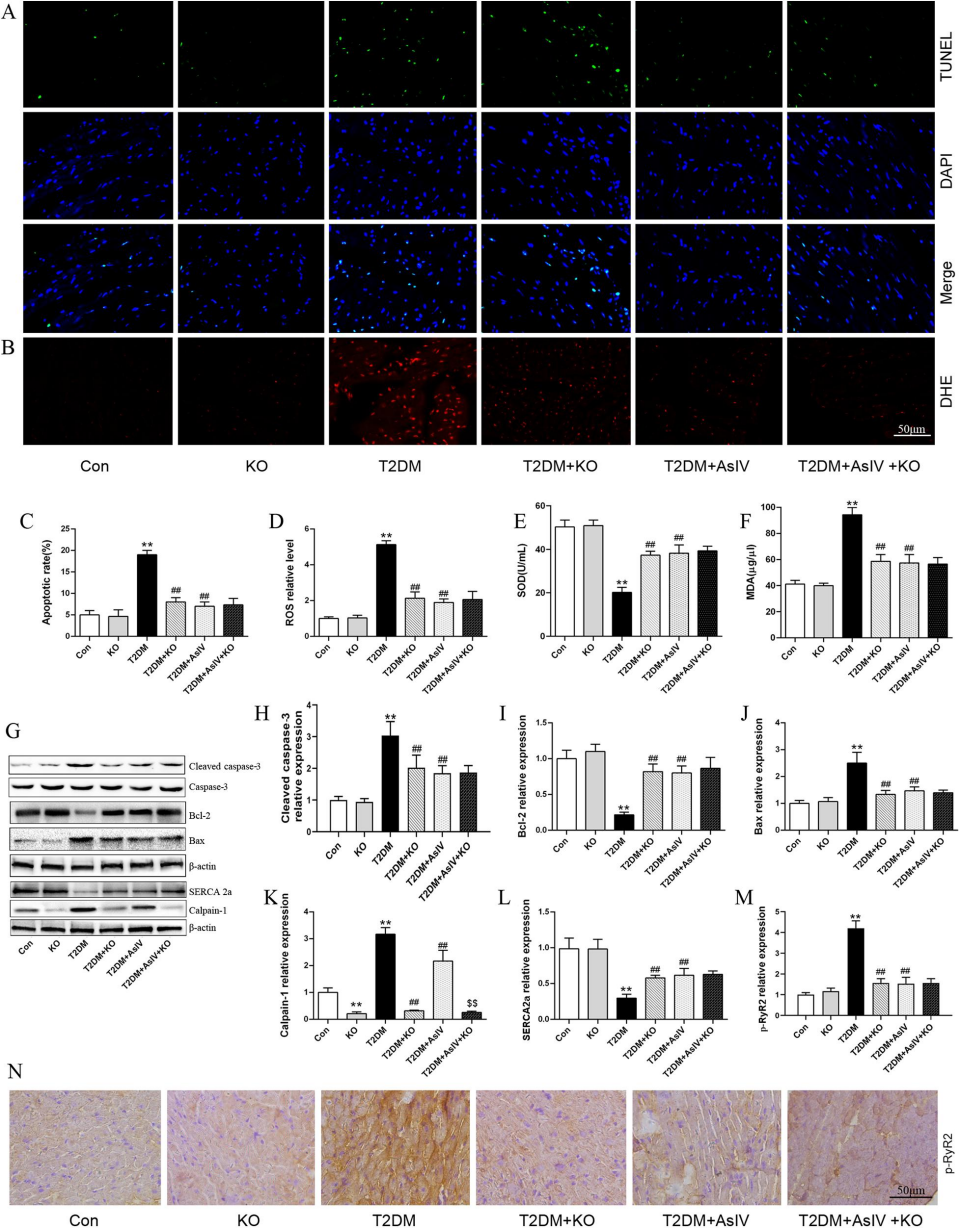
Astragaloside IV alleviates oxidative stress, apoptosis and Ca^2+^ metabolism imbalance through inhibiting calpain-1 in diabetes mice. **(A,C)** TUNEL staining and apoptotic rate (*n* = 4). **(B,D)** DHE staining and ROS level (*n* = 4). **(E,F)** The contents of MDA and SOD in serum (*n* = 8). **(G–L)** The protein expression of cleaved caspase-3, caspase-3, Bcl-2, Bax, SERCA2a and calpain-1 (*n* = 3). **(M,N)** Immunohistochemical staining and relative expression of p-RyR2 (*n* = 4). Data are expressed as the mean ± SD. *p < 0.05, **p < 0.01 vs. Con group; ^#^*p* < 0.05, ^##^*p* < 0.01 vs. T2DM group; ^$$^*p* < 0.01 vs. T2DM + AsIV group.

Compared with T2DM group, calpain-1 knockout decreased ROS level, MDA content, expression of bax and cleaved caspase-3, and increased SOD level and Bcl-2 expression, and the apoptosis rate of myocardial tissue cells decreased from 19.0% ± 1.1% to 8.0%±1.2% (Figures 6A–J). Compared with the group treated with AsIV alone, calpain-1 knockout did not further increase the inhibitory potency of AsIV on oxidative stress and apoptosis. Figure 6K revealed that the expression level of calpain-1 protein was markedly decreased in the KO group. Moreover, compared to the AsIV-treated group, the calpain-1 knockout further diminished the expression of calpain-1 (Figure 6K). Compared with the model group, the calpain-1 knockout group showed an increase in SERCA2a expression and decrease in p-RyR2 expression. Contrasted with the group treated with AsIV alone, calpain-1 knockout resulted in no further effect in p-RyR2 and SERCA2a expression (Figures 6L–N).

### 3.7 Astragaloside IV inhibited cardiomyocytes hypertrophy and cardiac fibroblasts proliferation

**Figure 7:**
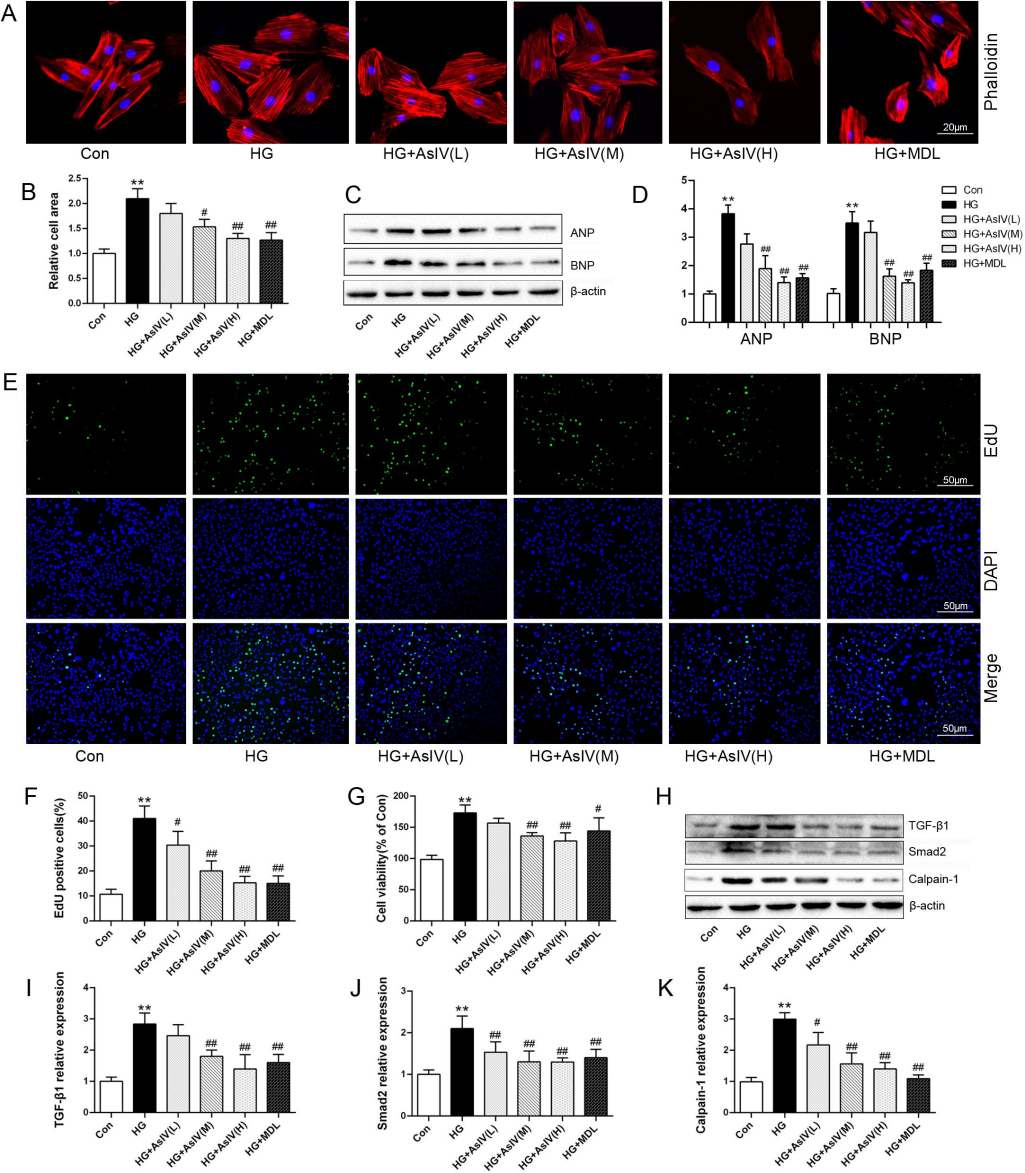
Astragaloside IV inhibited cardiomyocytes hypertrophy and cardiac fibroblasts proliferation. **(A,B)** Phalloidin staining and relative cell area of H9c2 cells. **(C,D)** ANP and BNP protein expression in H9c2 cells. **(E,F)** EdU staining of cardiac fibroblasts and the number of EdU positive cells. **(G)** Cell viability of cardiac fibroblasts. **(H–K)** Protein expression of TGF-β1, smad2 and calpain-1 in cardiac fibroblasts. Data are expressed as the mean ± SD (*n* = 4). *p < 0.05, **p < 0.01 vs. Con group; ^#^*p* < 0.05, ^##^*p* < 0.01 vs. HG group.

In the present investigation, the effects of AsIV on ventricular remodeling were assessed by examining H9c2 cardiomyocyte hypertrophy and fibroblast proliferation. High glucose treatment induced a 2.10 ± 0.20 fold expansion in cardiomyocyte area (Figures 7A,B) and significantly elevated the expression of the hypertrophic markers ANP and BNP (Figures 7C,D). Treated H9c2 cells with different concentrations of AsIV and MDL-28170 (the calpain-1 inhibitor) reduced the increase in myocardial H9c2 area and ANP and BNP protein expression caused by high glucose to varying degrees. EdU staining and CCK-8 were carried out to observe the effect of AsIV on the proliferation of cardiac fibroblasts. Compared with the control group, HG group showed significant proliferation of fibroblasts, manifested by increased green cell nuclei and increased cell viability (Figures 7E–G). Compared with the HG group, proliferation of fibroblasts decreased in the AsIV and MDL-28170 groups. In addition, WB results showed that high glucose led to an increase in calpain-1 protein expression in fibroblasts, as well as an increase in TGF-β1 and smad-2 proteins. AsIV inhibited the high glucose driven elevation in calpain-1, TGF-β1, and smad-2 protein expression (Figures 7H–K).

### 3.8 Astragaloside IV inhibited cardiomyocytes oxidative stress, apoptosis and Ca^2+^ overload

**Figure 8:**
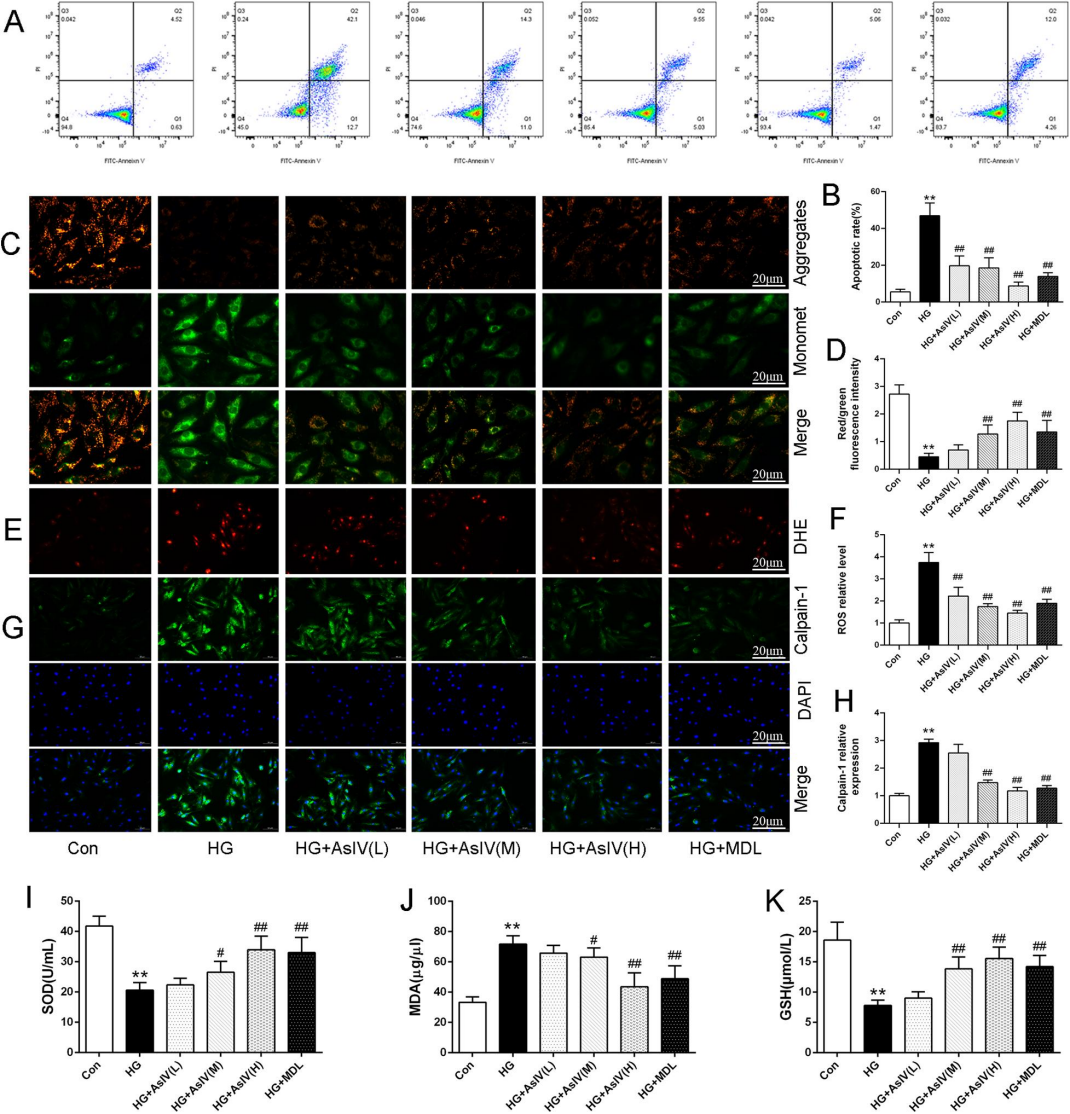
Astragaloside IV inhibited cardiomyocytes oxidative stress, apoptosis and Ca^2+^ overload. **(A,B)** Apoptosis image and apoptosis rate determined by flow cytometry. **(C,D)** JC-1 staining and MMP. **(E,F)** DHE staining and ROS level. **(G,H)** Immunofluorescence image of calpain-1 and relative expression of calpain-1 in H9c2 cells. **(I–K)** The content of SOD, MDA and GSH in cell supernatant. Data are expressed as the mean ± SD. (*n* = 3 for A-H and *n* = 8 for I-K). *p < 0.05, **p < 0.01 vs. Con group; *^#^p* < 0.05, ^##^*p* < 0.01 vs. HG group.

This study examined the effects of AsIV on oxidative stress, apoptosis, and Ca^2+^ overload in H9c2 cells. Flow cytometry analysis revealed a substantial increase in the apoptotic rate in the high glucose group compared with the control group (HG group: 46.9% ± 6.8%; Con group: 5.5% ± 1.5%). Nevertheless, treatment with AsIV was shown to alleviate apoptosis to different degrees (Figures 8A,B), and the apoptotic rates were 19.7% ± 5.4%, 18.6% ± 5.5% and 8.7% ± 2.1%, respectively. Mitochondrial membrane potential (MMP) was assessed using the JC-1 probe technique and quantified by relative fluorescence intensity as an early indicator of cell apoptosis. Consistent with apoptotic rate, decreased MMP caused by high glucose was reversed by AsIV (Figures 8C,D). As expected, the elevated ROS and MDA levels were reversed and the reduced SOD and GSH levels caused by high glucose was increased by AsIV and MDL-28170 (Figures 8E,F,I–K). Myocardial cells Ca^2+^ overload is closely related to calpain-1 expression, oxidative stress, apoptosis, and development of cardiac hypertrophy and remodeling. Intracellular Ca^2+^ level was evaluated by Fluo-3/AM probe in the current study. Compared with the Con group, both intracellular Ca^2+^ levels and calpain-1 expression were significantly increased in the HG group, and these elevations were reversed by high dose AsIV and the calpain inhibitor MDL-28170 (Supplementary Figure S3 and Figures 8G,H).

### 3.9 ASIV alleviates high glucose induced cardiomyocytes cells injury by inhibiting calpain-1 and CaMKII

**Figure 9:**
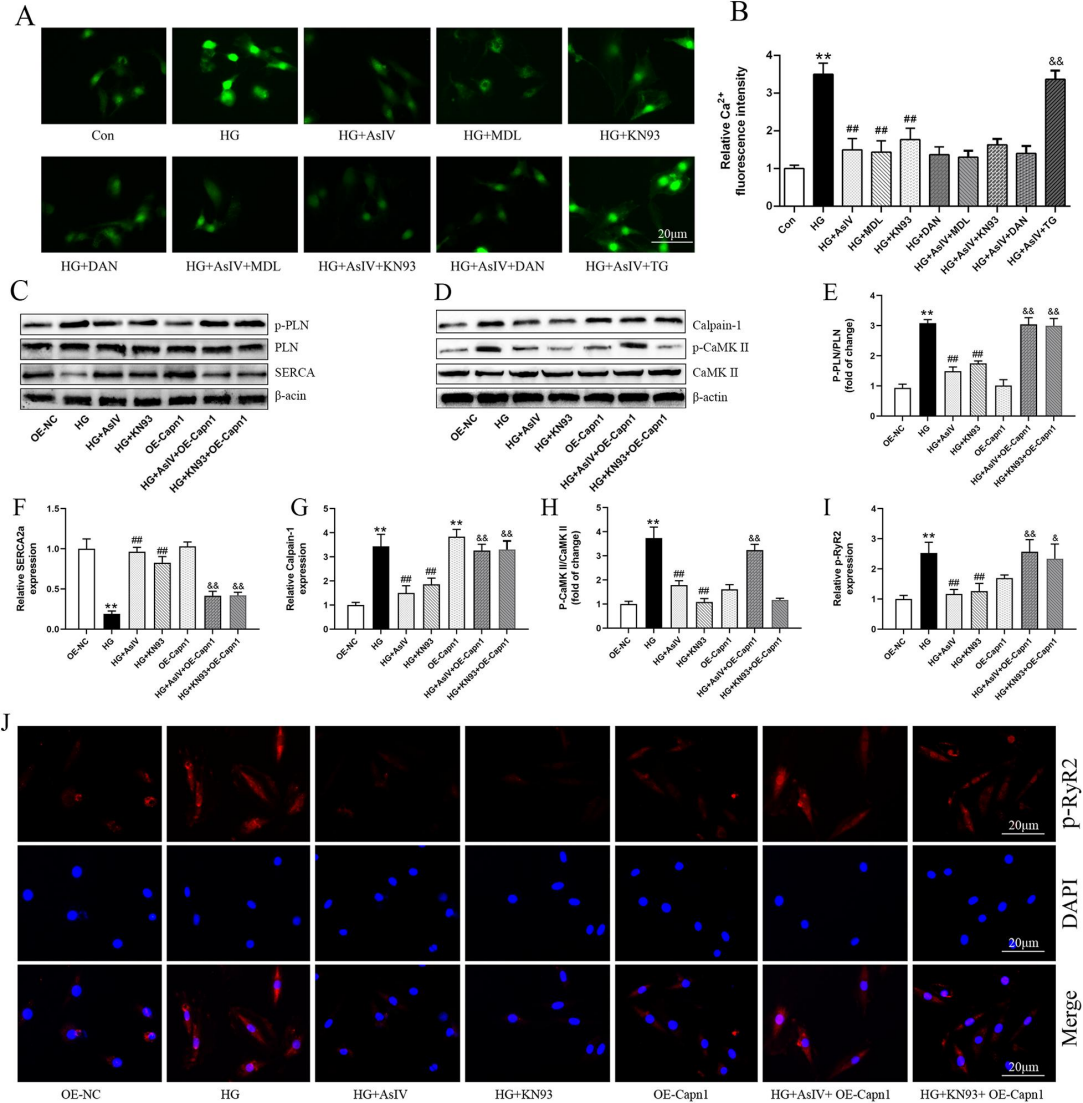
Astragaloside IV regulated Ca^2+^ dysregulation. **(A)** Ca^2+^ was measured by Fluo-3/AM probe. **(B)** The effect of AsIV(80 μM), KN93(10 μM), dantrolene(10 μM, DAN), and thapsigargin (1 μM, TG) on Ca^2+^ level. **(C–H)** The protein expression of SERCA2a, calpain-1, p-PLN and p-CaMKII. **(I,J)** p-RyR2 expression in H9c2 cells was determined by immunofluorescence. Data are expressed as the mean ± SD, (*n* = 3) *p < 0.05, **p < 0.01 vs. Con or OE-NC group; ^#^*p* < 0.05, ^##^*p* < 0.01 vs. HG group; ^&^*p* < 0.05, ^&&^*p* < 0.01 vs. HG + AsIV group or HG + KN93 group.

According to reports, intracellular Ca^2+^ overload can lead to myocardial cell hypertrophy and fibrosis through the CaMKII signaling pathway (28, 29). Therefore, in this study, we analyzed the p-CaMKII protein, and introduced the CaMKII inhibitor KN93, SERCA2a inhibitor thapsigargin, and RyR2 inhibitor dantrolene. Similar to the action of AsIV, KN93 and dantrolene inhibited the increase of Ca^2+^ induced by HG. AsIV combined with KN93 or dantrolene did not further inhibit the increase of Ca^2+^, but thapsigargin abolished the effect of AsIV on Ca^2+^ (Figures 9A,B). Calpain-1 was overexpressed using the lentiviral transduction method to explore the mechanism of action of AsIV. The results showed that overexpression of calpain-1 had no effect on the expression of p-PLN, SERCA2a and p-RyR2, but canceled the effects of AsIV and KN93 on the expression of p-PLN, SERCA2a and p-RyR2 (Figures 9C–I,J). Differently, overexpression of calpain-1 slightly increased the expression of p-CaMKII protein and canceled the inhibitory effect of AsIV on p-CaMKII, but could not cancel the inhibitory effect of KN93 on p-CaMKII. Similarly, KN93 partially reversed the high glucose induced increase in calpain-1 expression, while overexpression of calpain-1 canceled the inhibitory effects of KN93 and AsIV on calpain-1 expression (Figures 9D–H). To control for possible off-target effects of KN93, we repeated key experiments in the presence of its inactive analog KN92 (10 μM). KN92 neither reduced CaMKII phosphorylation at Thr286 nor reproduced the functional changes (p-RyR2^Ser2814^ and p-PLN^Thr17^) observed with KN93, confirming that the effects attributed to CaMKII inhibition are specific and not due to off-target actions of the inhibitor (Supplementary Figure S4).

### 3.10 AsIV alleviates high glucose induced cardiomyocytes cells injury

**Figure 10:**
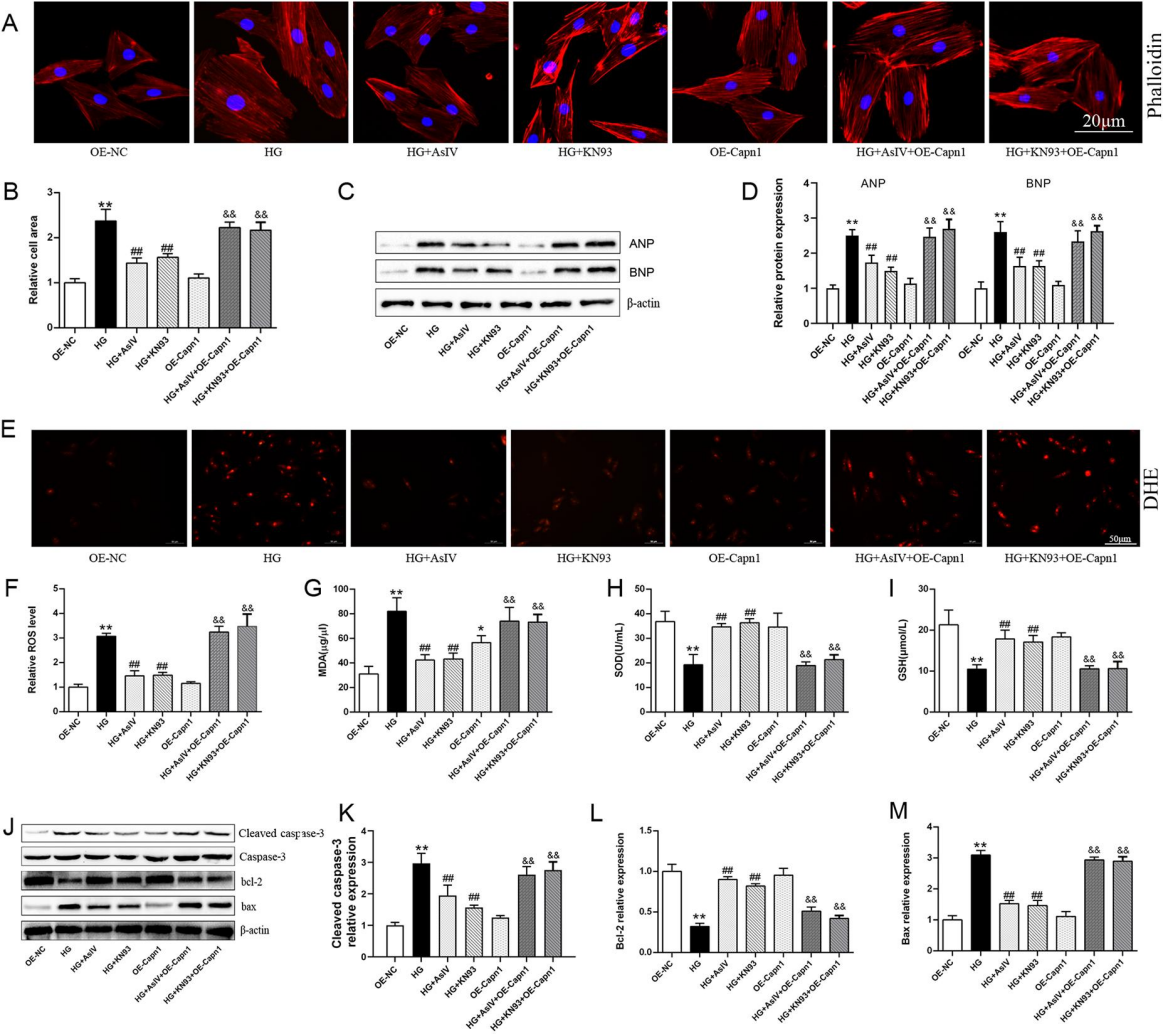
AsIV alleviates high glucose induced cardiomyocytes cells injury. **(A,B)** Relative cell area according to phalloidin staining (*n* = 4). **(C,D)** Protein expression of ANP and BNP (*n* = 3). **(E,F)** relative ROS level according to DHE staining (*n* = 4). **(G–I)** The content of SOD, MDA and GSH in cell supernatant (*n* = 8). **(J–M)** Protein expression of cleaved caspase-3, caspase-3, bax and bcl-2 (*n* = 3). Data are expressed as the mean ± SD. *p < 0.05, **p < 0.01 vs. OE-NC group; ^#^*p* < 0.05, ^##^*p* < 0.01 vs. HG group; ^&&^*p* < 0.01 vs. HG + AsIV group or HG + KN93 group.

Finally, the roles of calpain-1 and CaMKII in improving myocardial cell injury with AsIV were observed. The results showed that overexpression of calpain-1 alone did not cause myocardial cell enlargement, oxidative stress, or apoptosis. KN93 and AsIV have similar effects, inhibiting high glucose induced myocardial hypertrophy (Figures 10A–D), oxidative stress (Figures 10E–I), and apoptosis (Figures 10J–L). It is worth noting that the improvement effects of KN93 and AsIV on myocardial cell injury (hypertrophy, apoptosis, and oxidative stress) were all canceled by overexpression of calpain-1. Molecular docking result showed that AsIV stably interacted with CaMKII with docking energies lower than −5 kcal/mol (Supplementary Figure S5). These results showed that the protective effect of AsIV on myocardial cell injury was related with calpain-1 and CaMKII.

## 4 Discussion

Previous research has demonstrated that AsIV can ameliorate endothelial dysfunction caused by high glucose, and this beneficial effect may be associated with the regulation of calpain-1 (30). However, the role of calpain-1 in diabetic cardiomyopathy and whether AsIV ameliorates diabetic cardiomyopathy through the regulation of calpain-1 are still inconclusive. In the current study, calpain-1 knockout mice were constructed to observe the role of calpain-1 in diabetic myocardial hypertrophy and fibrosis. The effect of AsIV on diabetic cardiomyopathy was observed with metformin as a positive control drug. Due to limitations in experimental conditions, the E/è ratio of mice was not measured via ultrasonic examination in this study. However, results from the combination of E/A ratio, HR, EF, and FS indicated that AsIV, calpain-1 knockout, and metformin could improve both cardiac systolic and diastolic functions in diabetic mice. The results showed that T2DM induced an increase in the expression of calpain-1, and calpain-1 gene knockout or inhibition alleviated cardiomyocyte hypertrophy, fibrosis, intracellular Ca^2+^ overload, apoptosis and oxidative stress. AsIV showed a similar effect as calpain-1 inhibition, and calpain-1 overexpression abrogated the ameliorative effect of AsIV on myocardial injury at the level of H9c2 cells. As a classic hypoglycemic agent, metformin can lower blood glucose; however, its effect on improving already impaired cardiac function is slightly weaker than that of AsIV, and its inhibitory effect on the increased calpain-1 is also weaker than that of AsIV. This suggests that metformin's cardioprotective effect relies on blood glucose regulation, which is different from the direct cardioprotective effect of AsIV.

Calpain-1 is a key factor in diabetes and its associated cardiovascular complications. Teng et al. reported that endothelial cell-specific deletion of *Capns1* (disrupt calpain 1 and calpain 2) increased myocardial capillary density and coronary flow reserve contrast to their wild-type litter mates in type 1 and type 2 diabetes mice (12). The activity of calpain-1 and calpain-2 *in vivo* is tightly controlled by calpastatin. Overexpression of calpastatin or cardiomyocyte-specific knockout of Capn4 (regulatory subunit of calpain-1 and calpain-2) in mice inhibited the enhancement of cardiomyocyte cross-sectional areas and mRNA level of ANP and β-MHC induced by type 1 diabates and OVE26 mice (31). In the current study, we constructed mice with only calpain-1 knockout and established a mouse model of type 2 diabetes based on the knockout mice. Results showed that calpain-1 knockout reduced the increase of HW/BW and CAS, inhibited myocardial fibrosis, and improved cardiac systolic and diastolic dysfunction triggered by type 2 diabetes in mice. In addition, expression of calpain-1 dramatically increased in T2DM mice and HG induced H9c2 cells and myocardial fibroblasts, which effect was accompanied with impaired cardiac function, cardiac hypertrophy, and fibroblast proliferation in the current experiment. These findings, in conjunction with prior research, underscore the significance of calpain-1 as a potential therapeutic target in diabetic cardiomyopathy. However, the precise mechanisms underlying its involvement warrant further investigation.

Oxidative stress and apoptosis are of critical importance to the pathogenesis of diabetic cardiomyopathy. Oxidative stress, which arises from an imbalance between the generation of ROS and the body's antioxidant defenses, contribute significantly to diabetes-induced myocardial injury. Excessive ROS can inflict direct damage on cellular components such as lipids, proteins, and DNA, and trigger signaling pathways that promote cardiac hypertrophy and fibrosis. Apoptosis contributes to the loss of functional cardiomyocytes, leading to cardiac dysfunction and fibrosis. The present study demonstrates that both myocardial tissue from type 2 diabetic mice and high glucose treated H9c2 cells exhibit significantly elevated ROS levels, accompanied by reduced activity of SOD and GSH. Concurrently, we observed a marked increase in cardiomyocyte apoptosis, as evidenced by upregulated expression of pro-apoptotic Bax and cleaved caspase-3 along with downregulation of anti apoptotic Bcl-2, which collectively indicate activation of the mitochondria-dependent (intrinsic) apoptotic pathway under diabetic/high glucose conditions. Notably, calpain-1 knockdown *in vivo* and application of MDL-28170 *in vitro* unexpectedly reversed the above oxidative stress and apoptosis, suggesting that the damage of calpain-1 activation to cardiomyocytes may be related to oxidative stress and apoptosis. It was reported that transgenic calpastatin ameliorated mitochondrial oxidative stress and cell death, which contributed to improvement of cardiac function and myocardial remodeling in STZ mice (32). Importantly, the results indicated that AsIV inhibited calpain-1 expression, cardiomyocyte apoptosis, and oxidative stress while improving diabetic myocardial hypertrophy, fibrosis, and cardiac function. Calpain-1 knockout did not further increase the cardioprotective effect of AsIV, but calpain-1 overexpression abolished the effect of AsIV, confirming that the beneficial effect of AsIV on diabetic cardiomyopathy may be achieved by inhibiting calpain-1.

**Figure 11:**
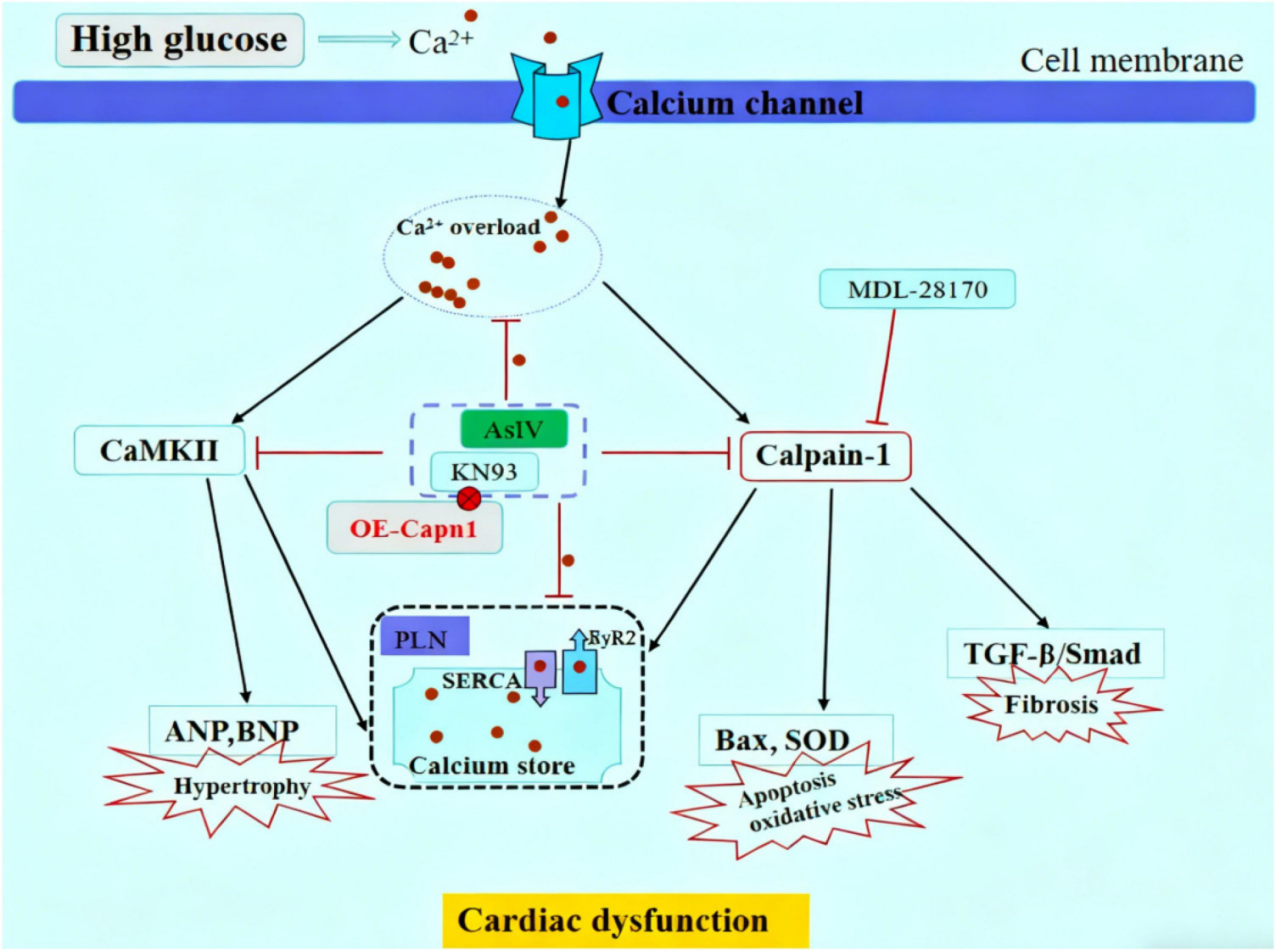
Schematic diagram of AsIV alleviating high glucose-induced cardiomyocyte injury via inhibiting Ca^2+^/calpain-1/CaMKII pathway.

Under hyperglycemic conditions, cytosolic Ca^2+^ level elevates resulting from impaired Ca^2+^ handling mechanisms, including downregulated SERCA2a activity and enhanced RyR2-mediated Ca^2+^ release as well as dysfunctional Na^+^/Ca^2+^ exchangers (33–37). Specifically, the reduced SERCA2a fails to efficiently pump cytosolic Ca^2+^ back into the sarcoplasmic reticulum (SR) for storage, while the hyperactive RyR2 promotes excessive Ca^2+^ leakage from the SR to the cytosol; together, these two alterations disrupt the dynamic balance of Ca^2+^ between the SR and cytosol. These changes lead to elevated cytosolic Ca^2+^ levels, which contribute to mitochondrial Ca^2+^ overload, oxidative stress, apoptosis, and activation of Ca^2+^-dependent cardiac hypertrophy signaling pathways (i.e., CaMKII and calcineurin/nuclear factor of activated T-cells signaling pathways) (38–41). Current studies have shown that the protective effect of AsIV is accompanied by a decrease in p-RyR2 and an increase in SERCA2a expression, along with reduced calpain-1 level and CaMKII phosphorylation. CaMKII, a major isoform of CaMK, is particularly implicated in DCM (42–44). Activated CaMKII phosphorylates target proteins such as RyR2 and phospholamban, further exacerbating Ca^2+^ leakage from the SR and impairing cardiac contractility. Results from present study showed that the expression of SERCA2a in myocardial tissue of diabetic mice was significantly decreased and p-RyR2 increased, while p-CaMKII protein was significantly increased. The same results were obtained at the H9c2 cell level, and intracellular Ca^2+^ overload was observed, which is in line with the above theory. In addition, in high glucose-challenged H9c2 cells, KN93 suppressed CaMKII phosphorylation and concurrently reduced p-PLN, p-RyR2 and SERCA2a expression, whereas its inactive analogue KN92 exerted no such effects, underscoring the pivotal role of CaMKII phosphorylation in high glucose-induced calcium mishandling. Calpain-1 and CaMKII interact in a variety of cardiovascular diseases and promote disease development (45, 46). The present study showed that calpain-1 activation slightly increased the expression of p-CaMKII protein and canceled the inhibitory effect of AsIV on p-CaMKII, while KN93 partially reversed the high glucose induced increase in calpain-1 expression, overexpression of calpain-1 canceled the inhibitory effects of KN93 and AsIV on calpain-1 expression. These findings highlight that the interaction between calpain-1 and CaMKII could represent a potential therapeutic target for AsIV in ameliorating diabetic cardiomyopathy (Figure 11).

## 5 Conclusion

To summarize, this study indicates that AsIV is capable of alleviating intracellular Ca^2+^ overload, apoptosis, and oxidative stress through regulation of the calpain-1/CaMKII signaling pathway. This mechanism contributes to the improvement of diabetes-induced myocardial hypertrophy and fibrosis. Nevertheless, there are limitations in our study. The interrelationship between calpain-1 and CaMKII signaling pathway should be further investigated with CaMKII genetically engineered mice and more advanced experimental methods.

## Data Availability

The original contributions presented in the study are included in the article/Supplementary Material, further inquiries can be directed to the corresponding author.
