# Supplementary material for: Astragaloside IV prevents calpain-1-mediated cardiac hypertrophy and fibrosis induced by diabetes
Source: Front Cardiovasc Med. 2026 Feb 5;13:1670472. doi: 10.3389/fcvm.2026.1670472 (PMC12916589; doi:10.3389/fcvm.2026.1670472)
Supplement: Supplementary file 1 [file Datasheet1.docx]

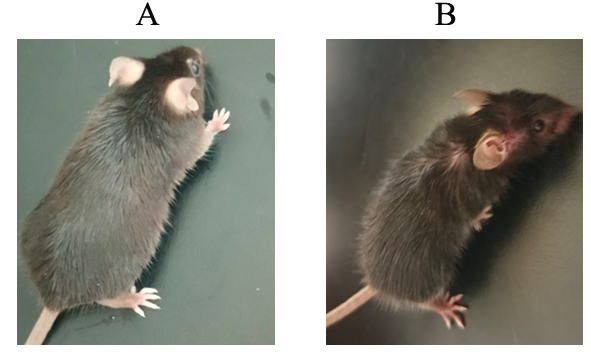


**Supplementary Figure 1.** A: Representative photos of mice in the normal group at Week 17. B: Representative photos of mice in the T2DM group at Week 17


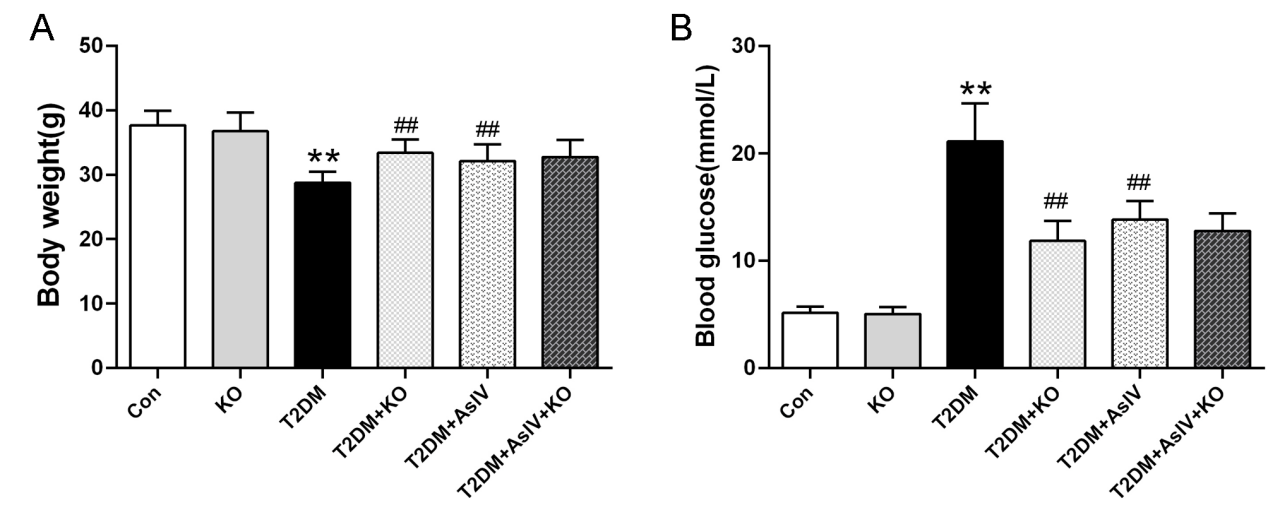


**Supplementary Figure 2.** (A)Mice body weight. (B) Mice blood glucose.(n =8 for A and B).^**^*p* < 0.01 vs. Con group; ^##^*p* < 0.01 vs. T2DM group;


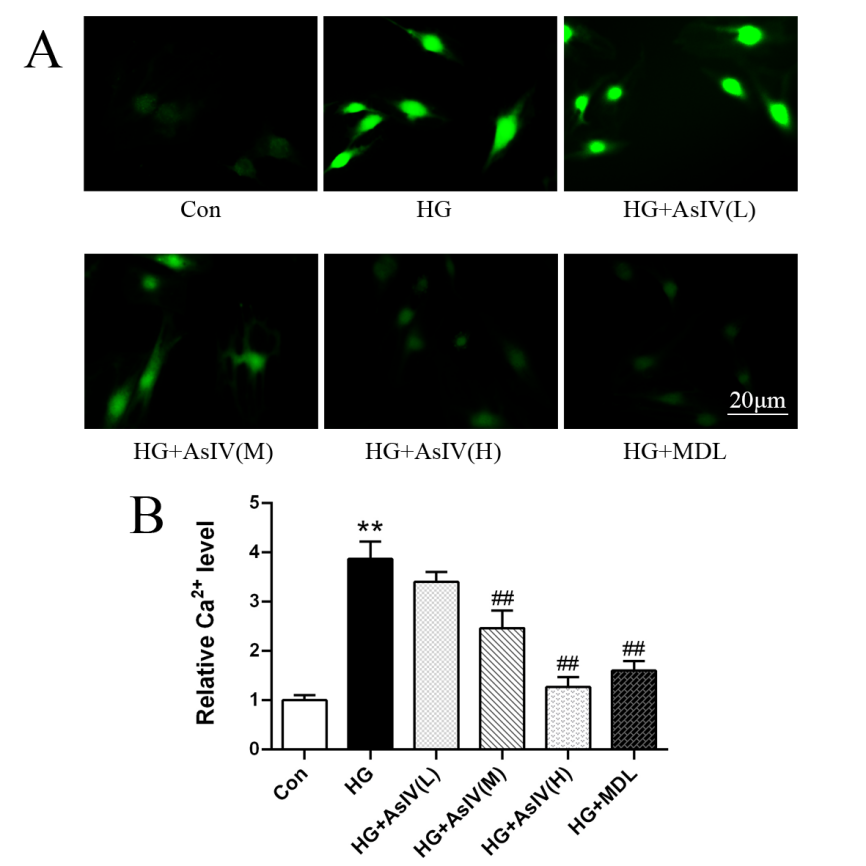


**Supplementary Figure 3.** (A) Intracellular Ca^2+^ was measured by Fluo-3/AM probe. (B) Relative Ca^2+^ level. Data were expressed as the mean ± SD. (n =3).***p* < 0.01 vs. Con; ^##^*p* <0.01 vs. HG group.


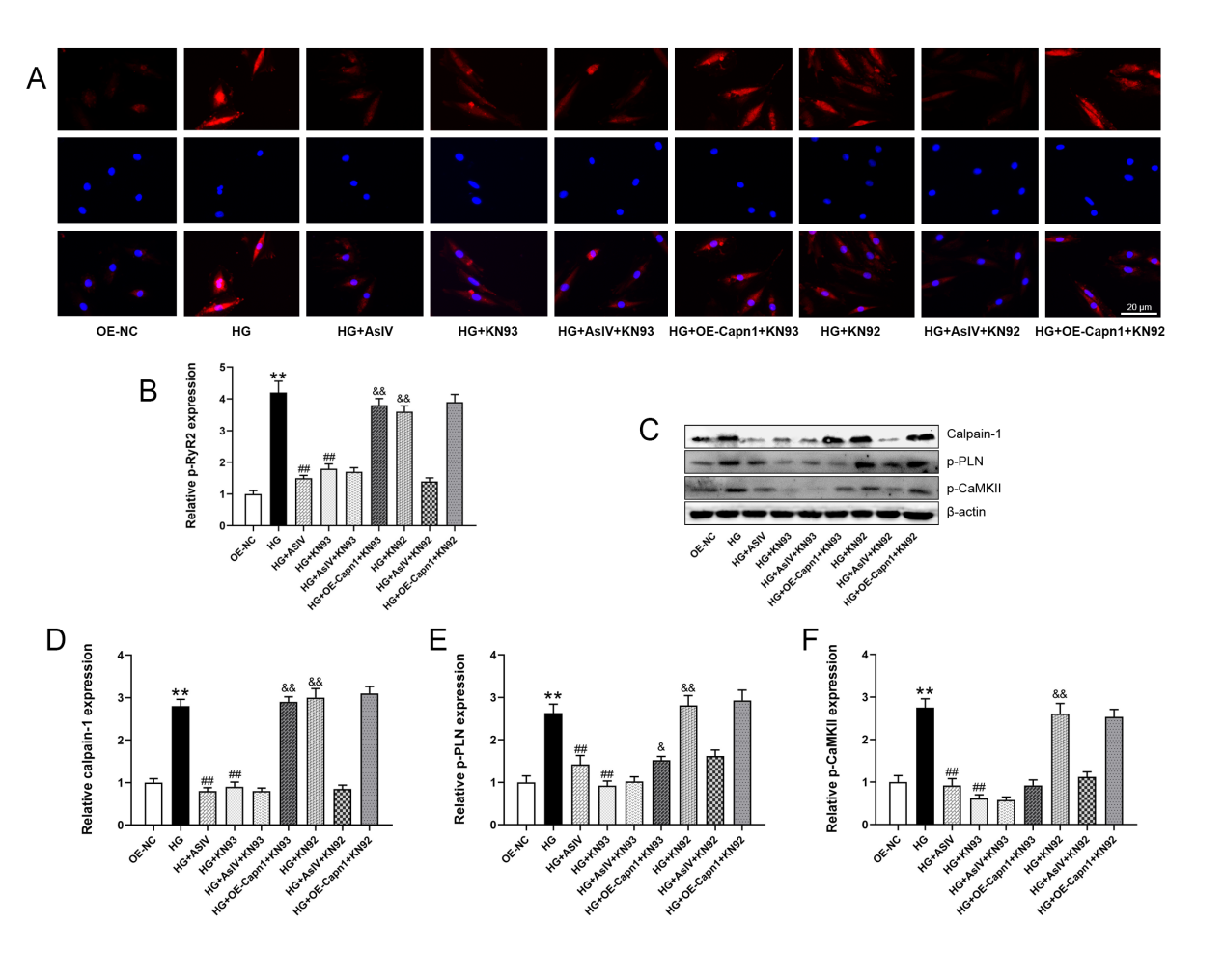


**Supplementary Figure 4.** (A-B) p-RyR2 expression in H9c2 cells was determined by immunofluorescence. (C-F): The protein expression of calpain-1, p-PLN and p-CaMKII. Data are expressed as the mean ± SD, (n=3). ^*^*p* <0.05, ^**^*p* < 0.01 vs. OE-NC group; ^#^*p* <0.05, ^##^*p* <0.01 vs. HG group; ^&^*p* <0.05, ^&&^*p* <0.01 vs. HG+KN93 group. ^$^*p* <0.05, ^$$^*p* <0.01 vs. HG+KN93 group.


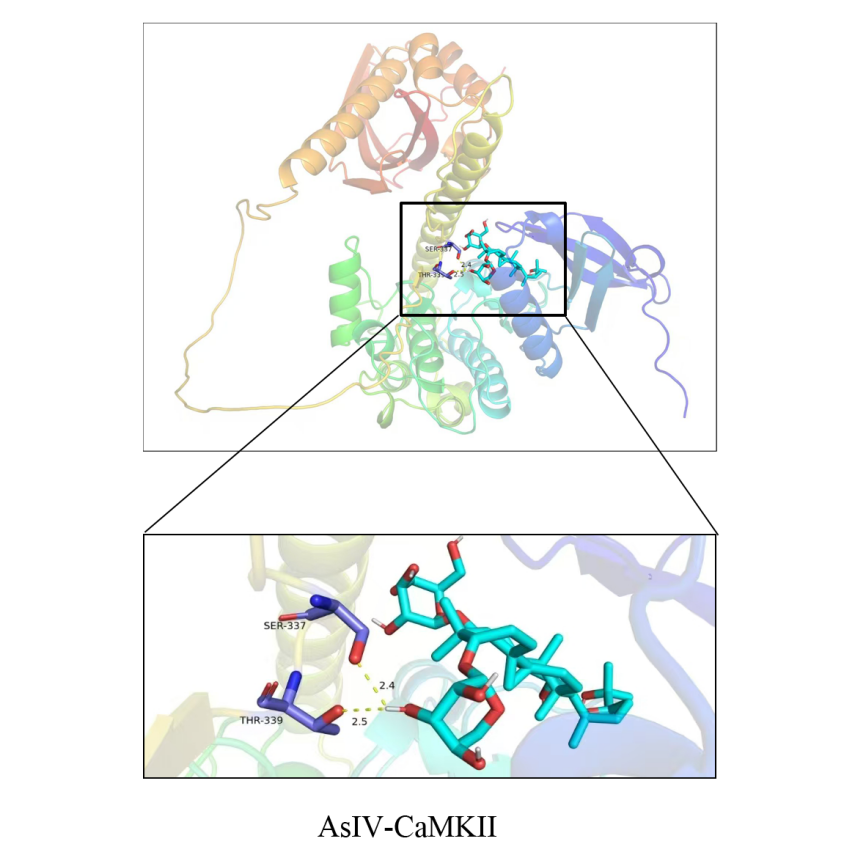


**Supplementary Figure 5.** Molecular docking results of AsIV with CaMKII.
